# A dermatological assessment of pediatric patients with tuberous sclerosis complex (TSC)^☆^

**DOI:** 10.1016/j.abd.2023.11.004

**Published:** 2024-04-23

**Authors:** Beatriz Azevedo Nunes, Ana Karolina Ferreira Gonçalves Romano, Mariana Aparecida Pasa Morgan, Alice Andrade Gonçalves, Laís Faria Masulk Cardozo, Luiz Gustavo Dufner de Almeida, Luciana Amaral Haddad, Ana Chrystina de Souza Crippa, Sergio Antonio Antoniuk, Kerstin Taniguchi Abagge

**Affiliations:** a Department of Pediatrics, Universidade Federal do Paraná, Curitiba, PR, Brazil; b Postgraduate Program in Child and Adolescent Health, Complexo Hospital de Clínicas, Universidade Federal do Paraná, Curitiba, PR, Brazil; c Department of Genetics and Evolutionary Biology, Centro de Pesquisa do Genoma Humano e Células-Tronco, Instituto de Biociências, Universidade de São Paulo, São Paulo, SP, Brazil

**Keywords:** Genetic diseases, Neurocutaneous syndromes, Tuberous sclerosis complex, Tuberous Sclerosis Complex 1 gene, Tuberous Sclerosis Complex 2 gene

## Abstract

**Background:**

Tuberous sclerosis complex (TSC) is a multisystem neurocutaneous syndrome with variable phenotypes. Recent updates of TSC diagnostic criteria reaffirmed the defined genetic diagnostic criterion as the finding of a pathogenic DNA alteration in either *TSC1* or *TSC2* genes. It also slightly modified definite clinical diagnostic criteria. TSC-associated skin lesions in infancy are important clinical signs to select individuals with possible TSC for a closer clinical follow-up and genetic testing.

**Objective:**

To raise awareness of the updated TSC diagnosis criteria; to assess the frequency of skin lesions in TSC patients as well as the first dermatological presentation; and to associate the findings with either *TSC1* or *TSC2* mutations.

**Methods:**

Observational cross-sectional study. Clinical and genetic data were retrospectively collected from 37 TSC patients from a Brazilian University Hospital. Patients with skin signs were examined and prospectively assessed for 12 months.

**Results:**

The earliest cutaneous lesions were hypomelanotic macules, which together with angiofibromas were the most frequent dermatological lesions. The total pathogenic DNA alteration ratio between *TSC2* and *TSC1* genes was 8:1. The frequency of a *TSC2* pathogenic variant was 10-fold greater in the presence of ungual fibromas.

**Study limitations:**

Small sample and a limited number of patients with *TSC1* pathogenic variants.

**Conclusion:**

Clinicians should be knowledgeable about TSC updated diagnostic criteria. Patients need to be followed up by a multidisciplinary team and treated accordingly. Early detection of cutaneous lesions is important for TSC diagnosis. A significant association between *TSC2* gene pathogenic alterations and ungual fibromas is described.

## Introduction

Tuberous Sclerosis Complex (TSC) is a neurocutaneous syndrome with variable phenotypes and autosomal dominant inheritance. It is due to pathogenic DNA alterations in either *TSC1* (OMIM 605284) or *TSC2* (OMIM 191092) genes and is characterized by benign tumors (hamartomas) in distinct organs, mainly the brain, skin, heart, kidneys, and lungs.^1^, ^2^ The *TSC1* gene (9q34) encodes hamartin, and *TSC2* (16p13.3) codes for tuberin. Under physiological conditions, hamartin, tuberin and TBC1D7 (OMIM 612655) proteins form a cytoplasmic complex that suppresses cell growth by inhibiting the mechanistic Target of Rapamycin (mTOR).^1^, ^3^, ^4^ *TSC1* or *TSC2* bi-allelic loss of function impairs the inhibitory effect on the mTOR pathway, causing abnormal cell differentiation and growth, resulting in hamartomatous lesions (Table 1).^3^, ^4^, ^5^

**Table 1:** Major and minor criteria for the clinical diagnosis of TSC according to Northrup et al., 2021.^3^

Major criteria	Minor criteria
Hypomelanotic macules (≥ 3, at least 5-mm diameter)	‘Confetti’ skin lesions
Facial angiofibromas (≥ 3) or fibrous cephalic plaque	Dental enamel pits (≥ 3)
Ungual fibromas (≥ 2)	Intraoral fibromas (≥ 2)
Shagreen patch	Retinal achromic patch
Multiple retinal hamartomas	Multiple renal cysts
Multiple cortical tubers and/or radial migration lines	Non-renal hamartomas
Subependymal nodules (≥ 2)	Sclerotic bone lesions
Subependymal giant cell astrocytoma	
Cardiac rhabdomyoma	
Lymphangioleiomyomatosis^a^	
Angiomyolipomas (≥ 2)^a^	

Underlined terms highlight updated criteria.^3^

Definite TSC clinical diagnosis: two major features or one major plus two or more minor features.

Possible TSC clinical diagnosis: one major criterion or at least two minor criteria.

a Combination of these two major criteria without other features does not meet the criteria for a definite diagnosis.

Since 2012 the presence of a pathogenic alteration in *TSC1* or *TSC2* is a definite and sufficient criterion to diagnose TSC, regardless of the clinical findings.^4^ In 2021, the genetic diagnostic criterion was reaffirmed, and the combination of clinical criteria leading to definite or possible clinical diagnoses was maintained (Table 1).^3^ There were however few changes in the clinical criteria. Multiple cortical tubers and/or radial migration lines have replaced the general term cortical dysplasia as a major criterion, and sclerotic bone lesions were reinstated as a minor criterion (Table 1).^3^ Patients with definite, possible, or suspected TSC diagnosis should be followed up by a multidisciplinary team for the early identification of signs and symptoms, clinical management, and therapeutic intervention, resulting in better clinical outcomes.^3^, ^4^

The association between TSC genotypes and phenotypes has been studied, highlighting *TSC2* alterations more frequently among patients with earlier presentations of seizures, the presence of West syndrome (epileptic/infantile spasms with onset in infancy or early childhood associated with hypsarrhythmia and developmental regression), and more severe cognitive deficit.^2^ Although genotype-phenotype analyses mainly focus on neurological and renal signs, which are the major causes of morbidity and mortality,^3^ dermatological signs are one of the earliest clinical presentations, and their detection can lead to suspected TSC.^5^, ^6^

TSC has been often underdiagnosed notably in developing countries.^7^ The early onset of some cutaneous signs is highly suggestive of TSC (Table 1). Given their importance to the diagnosis and treatment of this neurocutaneous genetic disorder, this study aimed at characterizing the skin lesions of 37 patients with definite clinical and genetic diagnoses of TSC. The authors additionally classified patients according to the gene involved and verified in each gene category the frequency of specific skin lesions. The authors describe in the TSC cohort a significant association between ungual fibromas and TSC2 alterations.

## Subjects and methods

This observational, analytical and cross-sectional study was conducted at the Department of Pediatrics of the Clinics Hospital Complex of the Federal University of Paraná (CHC-UFPR), Curitiba, Brazil. The study protocol was approved by the CHC/UFPR Research (CAAE no. 67137317.5.0000.0096) and the University of São Paulo (CAAE no. 12572913.3.3002.5479 and 48259715.2.3003.5505) Ethics Committees.

Patients included in the study were clinically diagnosed with TSC according to Northrup et al. (2013)^4^ and had a pathogenic DNA variant previously identified by Sanger, Multiplex Ligation-dependent Probe Amplification (MLPA) or Next-Generation Sequencing (NGS) in either *TSC1* (NM_000368.4) or *TSC2* (NM_000548.3) genes at the University of São Paulo, São Paulo, Brazil (data not shown). After the publication of the updated diagnostic criteria (Northrup et al., 2021),^3^ data were reviewed accordingly and no alteration was made. In this ambispective study, once the family had provided written informed consent, the medical records were retrospectively reviewed. Then patients were prospectively seen by the Pediatric Dermatology team during a joint effort for the care of TSC patients in Oct/2021 and Oct/2022. Information (identification, age, dates, genetic study result, and description of the skin lesions) was registered and clinical lesions were photographed. Data were statistically analyzed in Excel®.

The sample was non-probabilistically and systematically selected by convenience, in order of appointment and admission to the study. Measures of central tendency and dispersion were expressed as mean ± standard deviation for continuous variables with symmetrical distribution and median (Interquartile Range [IQR]) for those with asymmetrical distribution. Categorical variables were expressed as absolute and relative frequencies. The Mann-Whitney non-parametric test was used to estimate the difference between continuous variables with asymmetric distribution and the Fisher’s exact and Pearson’s Chi-Square tests were used to estimate the difference between categorical variables. All tests considered a minimum significance level of 5%, 10% type II error, and magnitude of the effect of estimated difference between mutated genes four times.

## Results

The study sample comprised a total of 37 patients (36 unrelated patients), with definite clinical and genetic diagnoses of TSC, including 20 (54.1%) boys and 17 (45.9%) girls. All of them had at least one dermatological sign considered as a TSC diagnostic criterion (Table 1). All patients had first skin lesion onset before 18 years of age. The median age at TSC diagnosis was 18 months (IQR: 8‒60), and the median age at dermatologic evaluation was 16 years (IIQ: 9‒24.5).

Twenty-seven families could inform the first cutaneous sign identified in the patient. The patients’ median age at its onset was 3 months (IQR: 0‒19), and the most frequent skin lesion first identified was hypomelanotic leaf macula (25/27; p < 0.001). Considering patients according to sex, no difference was observed in first cutaneous signs, age at its onset, age at TSC diagnosis, or mutated gene (p > 0.05; Table 2).

**Table 2:** Age at onset of first skin lesion, TSC diagnosis, and first cutaneous signs according to sex.

Age and skin lesion	Female (n = 14)	Male (n = 13)	p
	[Median (IIQ); n (%)]	[Median (IIQ); n (%)]	
Median age at first skin sign onset (months)	2.0 (0‒19)	3.0 (0‒7)	0.92^a^
Median age at TSC diagnosis (months)	18.0 (12‒36)	24.0 (7‒60)	0.78
First cutaneous sign			
Hypomelanotic leaf macula	12 (92.9%)	13 (92.3%)	1.00^b^
Confetti-like lesion	0 (0.0%)	2 (15.4%)	0.22^b^
Fibrous cephalic plaque	2 (14.3%)	1 (7.7%)	1.00^b^
Intraoral fibrous plaque	0 (0.0%)	1 (7.7%)	0.48^b^
Gene harboring pathogenic alteration			
*TSC1*	4 (20.0%)	1 (5.9%)	0.34^b^
*TSC2*	16 (80.0%)	16 (94.1%)	

a Mann-Whitney test.

b Fisher’s exact test, n = 27.

**Figure 1:**
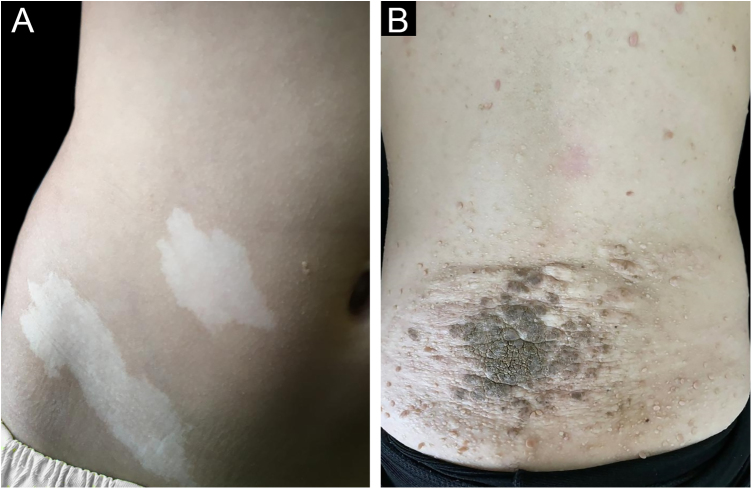
(A‒B) Hypomelanotic macules on the patient’s abdomen (A) and shagreen plaque on the back of an adolescent patient (B).

**Figure 2:**
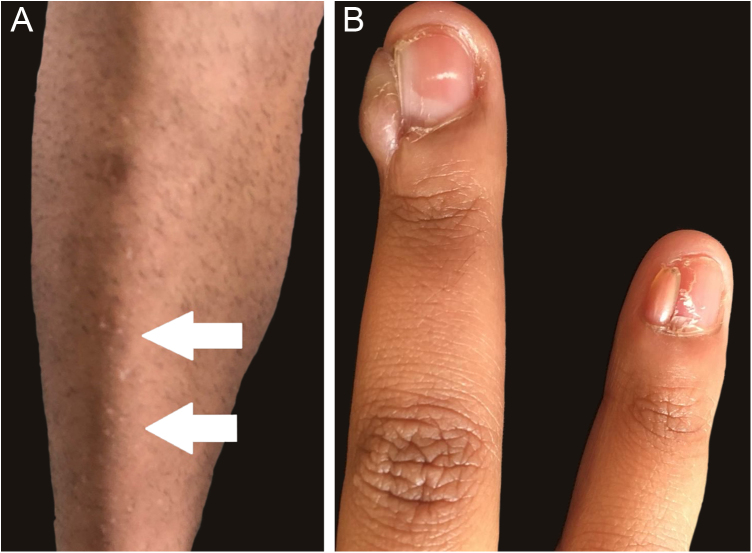
(A‒B) Confetti-like lesions (arrows) in the anterior tibial region (A) and ungual fibromas in 4^th^ and 5^th^ fingers of an adult patient (B).

**Figure 3:**
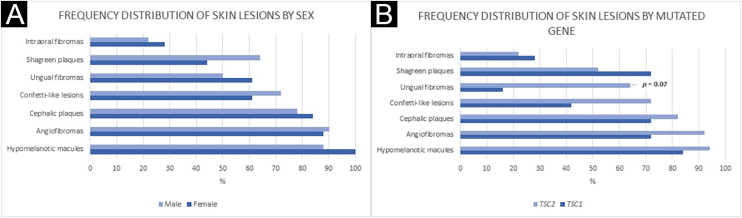
Frequency distribution of skin lesions by sex (A) and mutated gene (B). (A) Fisher’s exact test: Hypomelanotic macules: p = 0.23; Angiofibromas: p = 1.00; Cephalic plaques: p = 0.70; Confetti-like lesions: p = 0.50; Ungual fibromas: p = 0.53; Shagreen plaques: p = 0.33; Intraoral fibromas: p = 0.73. (B) Fisher’s exact test: Hypomelanotic macules: p = 0.44; Angiofibromas: p = 1.00; Cephalic plaques: p = 0.61; Confetti-like lesions: p = 0.18; Ungual fibromas: p = 0.07; Shagreen plaques: p = 0.42; Intraoral fibromas: p = 1.00.

Figure 1, Figure 2 illustrate some of the main cutaneous findings in the TSC patients from this cohort, which were hypomelanotic macules (92.5%), angiofibromas (87.5%), cephalic plaques (80%), confetti-like lesions (67.5%), shagreen patches (55%), ungual fibromas (55%), and intraoral fibromas (25%). When each TSC skin lesion was individually considered there was no difference between sexes (p > 0.05; Fig. 3A). The number of angiofibromas was also similar between sexes (p = 0.31). Data collected from 37 patients (median age 16 [4‒41] years) that had been examined at two time points (Oct/2021, Oct/2022) disclosed no new skin lesion one year later.

All 37 TSC patients had DNA assessed by sequencing, including two siblings. The 36 pathogenic DNA alterations were found in the *TSC1* gene in four unrelated individuals (11.1%) and in *TSC2* in 32 (88.9%) patients, defining a *TSC2*:*TSC1* mutation ratio of 8:1. There were three frameshifting variants and one large deletion in *TSC1*. The two siblings had a *TSC1* pathogenic mutation, summing up five individuals with alterations in this gene. *TSC2* mutations comprised seven frameshiftings, seven unique missense, six unique nonsense, three splicing, and three in-frame deletion variants, plus three large deletions and one large duplication. One *TSC2* nonsense variant was found in three unrelated patients, totaling 32 patients with *TSC2* mutations.

The frequencies of *TSC1* and *TSC2* pathogenic alterations were similar between sexes (Table 2; p = 0.34). Age at first skin lesion onset and age at TSC diagnosis were available for 5 and 22 patients with *TSC1* and *TSC2* pathogenic DNA alterations, respectively. There were no significant differences between patient groups with distinct altered genes when comparisons were made for ages at first skin lesion, at TSC diagnosis, or type of first skin lesion (Table 3; p > 0.05).

**Table 3:** Age at onset of first skin lesion, TSC diagnosis, and total cutaneous signs according to the gene harboring the pathogenic alteration.

Age, diagnosis and number of cutaneous signs	*TSC1* (n = 5)	*TSC2* (n = 32)	p
	[Median (IIQ); n (%)]	[Median (IIQ); n (%)]	
Age and skin lesion			
Median age at first skin sign onset (months)	0.0 (0‒18)^a^	3.5 (0‒19)^b^	0.39^c^
Median age at TSC diagnosis (months)	30.0 (15‒57)^a^	15.0 (8‒60)^b^	0.80^c^
Number of observed cutaneous signs			
Hypomelanotic leaf macula	4 (80.0%)	31 (96.9%)	0.25^d^
Angiofibromas	5 (100.0%)	32 (100.0%)	1.00^d^
Fibrous cephalic plaque	4 (80.0%)	26 (81.2%)	1.00^d^
Confetti-like lesion	3 (60.0%)	23 (71.9%)	0.62^d^
Shagreen patches	4 (80.0%)	18 (56.2%)	0.62^d^
Ungual fibromas	1 (20.0%)	21 (65.6%)	0.13^d^
Intraoral fibromas	1 (20.0%)	8 (25.0%)	1.00^d^

a n = 5.

b n = 22.

c Mann-Whitney test.

d Fisher’s exact test.

**Figure 4:**
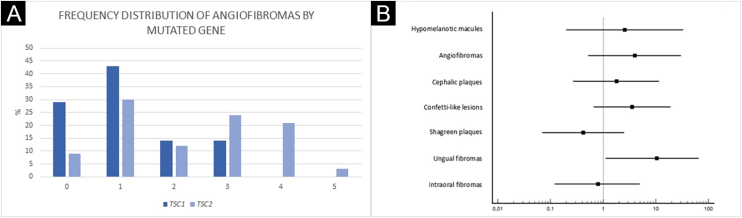
Angiofibromas by mutated gene (A) and Odds Ratio of TSC skin lesions for *TSC2* mutations (B). (A) Pearson’s Chi-Square test: p = 0.64. (B) OR, Odds Ratio; Ungual fibromas: OR = 10.50; p = 0.03.

Ungual fibromas were more frequent among patients with *TSC2* than *TSC1* genetic alteration (Fig. 3B; 65.6% vs. 20.0%, for *TSC2* vs. *TSC1*; p = 0.07). The odds ratios estimated for specific skin lesions disclosed a ten-fold increase for ungual fibroma when a *TSC2* pathogenic alteration was present (Fig. 4A; Odds Ratio = 10.50, 95% Confidence Interval: 1.12–67.21, p = 0.03). The number of angiofibromas per patient was similar between patients harboring a pathogenic alteration in different genes (Fig. 3B; p = 0.64) and varied from zero to five (Fig. 4B).

## Discussion

TSC-associated skin lesions should be recognized by general practitioners and pediatricians as they are commonly the first noticeable signs of this disease and comprise four of the eleven major criteria and three of the seven minor criteria for the clinical diagnosis of TSC (Table 1). Early diagnosis is important for follow-up and appropriate treatment initiation, limiting the growth of lesions and related morbidity. MTOR inhibitors received clinical approval as oral therapy for specific TSC lesions, such as everolimus to treat subependymal giant cell astrocytoma,^8^ renal angiomyolipoma,^9^ and as adjunctive treatment of TSC-partial onset seizures^10^; and sirolimus (rapamycin) for lymphangioleiomyomatosis.^11^ In clinical studies, topical sirolimus decreases angiofibromas, hypomelanotic macules, or cephalic plaques, and is well tolerated by children.^12^, ^13^, ^14^ Therefore, TSC skin lesions are not only important for the diagnosis of the disease but can be effectively treated by targeting mTOR hyperactivation.

In this cohort of 37 patients with definite clinical and genetic diagnoses of TSC, the most common initial skin sign was hypomelanotic macules, which were also the most frequent dermatological presentation (92.5%). Hypomelanotic macules occur in approximately 90% of patients and have their onset usually at birth or in early childhood,^6^ as observed (n = 27 informative cases; mean age of 3 months; Table 2). Other first skin signs less often observed were confetti-like lesions, fibrous cephalic or intraoral fibrous plaques (Table 2). Among one-month-old infants with at least one suggestive sign of TSC, hypomelanotic macules were the cause of the diagnosis suspicion in 31% of them.^15^ In patients up to four months of age, hypomelanotic macules were the initial sign in 35%.^7^ The present study did not assess non-dermatological signs. It confirms that among skin lesions hypomelanotic macules are the most frequent first sign in TSC patients (Table 2).

Facial angiofibromas were the second most frequent lesion (87.5%), followed by cephalic plaques (80%). Overall angiofibroma estimated occurrence (75%)^6^ rises in the second decade of life upon puberty.^7^ Thus, angiofibromas’ frequency depends on the mean age of the patients at enrollment in the cohort. Shagreen plaques are generally observed from the first decade of life on, more commonly after five years of age,^5^, ^16^ in a frequency (50%) similar to the one observed by us (55%). Confetti-like lesions were detected in 67.5% of patients. They usually start in the first decade of life but have limited usefulness in adults as a diagnostic criterion, as they can develop as a consequence of chronic sun exposure.^6^ A history of onset in early childhood is suggestive of TSC.^5^ Intraoral fibromas occur in 20%–50% of patients, more frequently in adults than in children.^6^ Accordingly, the authors detected these lesions in ten TSC patients (25%). Skin lesion distribution was not different between sexes (Table 2). Gingival fibromas have been identified more frequently among males than females.^17^

The mean age at diagnosis was 18 months in this sample, 15 months later than the mean age at first skin sign detection, and later than the mean age at diagnosis from a recent study (n = 86; 6 months) that also adopted the same diagnostic criteria.^4^ In this German study, cardiac rhabdomyoma, a major TSC clinical criterion (Table 1), detected by prenatal fetal imaging contributed to diagnosing earlier the disease in 22% of cases.^6^ It appears that patients in the Brazilian cohort have heterogeneous access to fetal imaging analyses and depend more commonly on tertiary referral hospitals for the establishment of the TSC diagnosis.

Genetic testing is useful to confirm the diagnosis of TSC, particularly in clinically challenging cases in the early stages. In this analysis, *TSC1* and *TSC2* pathogenic variants were observed in 13.5% (5/37) and 86.5% (32/37) of patients, respectively, reasonably above the ratio observed by other studies.^17^, ^18^, ^19^, ^20^, ^21^ The predominance of *TSC2* pathogenic genetic alterations, eight times more common than *TSC1* pathogenic variants, was observed among females (8-fold among females and 3.4-fold among males; Table 2). The chance of a *TSC2* mutation was 10-fold higher in the presence of ungual fibromas, although these data should be viewed with caution because of the wide confidence interval associated with the small number of cases with a *TSC1* mutation.

A more severe neurological phenotype may be associated with *TSC2* mutation,^17^, ^18^, ^19^, ^20^, ^21^ while skin lesions and genotype association appear more variable among studies, except for facial angiofibromas. The TSC natural history database disclosed a significant association between *TSC2* mutation and angiofibromas.^22^ The mean grade of facial angiofibroma appears to be higher in patients with a *TSC2* than *TSC1* alteration.^18^ The authors did not observe a difference in the number of angiofibromas, which could be due to the low number of patients with a *TSC1* mutation, although there appears to be a trend of increasing numbers of angiofibromas in the presence of a *TSC2* gene mutation (Fig. 4A). In another study, children at age of two years with a *TSC1* gene mutation had less hypomelanotic macules and facial angiofibromas than those with a *TSC2* genetic alteration.^23^ By contrast, Shagreen patches have been observed more frequently in patients with a *TSC1* than *TSC2* gene alteration.^17^ Among 38 TSC patients with at least one cutaneous sign, dermatological examination alone was sufficient to establish a definite diagnosis of the disease in 34, demonstrating the importance of early recognition of skin lesions.^24^, ^25^

The TSC cohort from a reference clinical center in Southern Brazil discloses the association between ungual fibromas and *TSC2* genetic alteration. One limitation is the small sample size and the limited number of patients with *TSC1* pathogenic variants. Further studies are necessary to evaluate TSC dermatological presentation association with genotype. The authors confirm the importance of detecting skin lesions for early identification of TSC. Clinicians should be aware of TSC's possible mucocutaneous lesions, their variable expressivity, and the natural history of the disease.^26^

## Financial support

This research was supported by São Paulo Research Foundation (FAPESP) grants 2013/08028-1 and 2019/10868-4 (São Paulo, Brazil), and CAPES (Process:88881.132401/2016-01; Brasília, Brazil).

## Authors’ contributions

Beatriz Azevedo Nunes: Study concept and design; data collection, analysis and interpretation; statistical analysis; writing of the manuscript; effective participation in the research guidance; critical review of the literature; and final approval of the final version of the manuscript.

Ana Karolina Ferreira Gonçalves Romano: Study concept and design; data collection, analysis and interpretation; statistical analysis; writing of the manuscript; effective participation in the research guidance; critical review of the literature; and final approval of the final version of the manuscript.

Mariana Aparecida Pasa Morgan: Study concept and design; analysis and interpretation of data; statistical analysis; writing of the manuscript; effective participation in the research guidance; critical review of the literature; and final approval of the final version of the manuscript.

Alice Andrade Gonçalves: Data collection; writing of the manuscript; effective participation in the research guidance; critical review of the literature; and final approval of the final version of the manuscript.

Laís Faria Masulk Cardozo: Study concept and design; data collection; effective participation in the research guidance; critical review of the literature; and final approval of the final version of the manuscript.

Luiz Gustavo Dufner de Almeida: Study concept and design; data collection, analysis and interpretation; statistical analysis; effective participation in the research guidance; critical review of the literature; and final approval of the final version of the manuscript.

Luciana Amaral Haddad: Study concept and design; analysis and interpretation of data; statistical analysis; writing of the manuscript; effective participation in the research guidance; critical review of the literature; and final approval of the final version of the manuscript.

Ana Chrystina de Souza Crippa: Study concept and design; intellectual participation in the propaedeutic and/or therapeutic conduct of the studied cases.

Sergio Antonio Antoniuk: Study concept and design; intellectual participation in the propaedeutic and/or therapeutic conduct of the studied cases.

Kerstin Taniguchi Abagge: Study concept and design; analysis and interpretation of data; statistical analysis; effective participation in the research guidance; intellectual participation in the propaedeutic and/or therapeutic conduct of the studied cases; critical review of the literature; and final approval of the final version of the manuscript.

## Conflicts of interest

None declared.
